# The association between lipid biomarkers and osteoarthritis based on the National Health and Nutrition Examination Survey and Mendelian randomization study

**DOI:** 10.1038/s41598-024-51523-8

**Published:** 2024-01-16

**Authors:** Guoxin Huang, Xian Zhong, Meiling Zhang, Ming Xu, Bin Pei, Da Qian

**Affiliations:** 1 Department of Evidence-Based Medicine Center, Xiangyang No.1 People’s Hospital, Hubei University of Medicine, Xiangyang, 441000 China; 2 Department of Burn and Plastic Surgery-Hand Surgery, Changshu Hospital Affiliated to Soochow University, Changshu No.1 People’s Hospital, Changshu, 215500 China; 3 Department of the second ward of Orthopedic, Xiangyang No.1 People’s Hospital, Hubei University of Medicine, Xiangyang, 441000 China

**Keywords:** Computational biology and bioinformatics, Genetics, Medical research, Pathogenesis, Risk factors

## Abstract

To explore the association between lipid markers and osteoarthritis (OA). First, the National Health and Nutrition Examination Survey (NHANES) database was used to screen participants with lipid markers, OA and relevant covariates, and logistic regression was used to analyze the association between lipid markers and OA; Then, under the theoretical framework of Mendelian randomization (MR), two-sample MR was performed using GWAS data of lipid markers and OA to explore the causal association between the two, which was analyzed by inverse variance weighting (IVW) method. Heterogeneity test, sensitivity analysis and pleiotropy analysis were also performed. The NHANES database screened a total of 3706 participants, of whom 836 had OA and 2870 did not have OA. When lipid markers were used as continuous variables, multivariate logistic results showed an association between HDL, LDL and OA (HDL, OR (95%):1.01 (1.00, 1.01); LDL, OR (95%):1.00 (0.99, 1.00)). When lipid markers were used as categorical variables, multivariate logistic results showed the fourth quartile result of 0.713 (0.513, 0.992) for LDL relative to the first quartile. In MR study, the results of the IVW method for TG, TL, HDL and LDL showed OR (95% CI) of 1.06 (0.97–1.16), 0.95 (0.85–1.06), 0.94 (0.86–1.02) and 0.89 (0.80–0.998) with P-values of 0.21, 0.37. 013, 0.046. The heterogeneity tests and multiplicity analyses showed P-values greater than 0.05, and sensitivity analyses showed no abnormal single nucleotide polymorphisms. Through NHANES database and MR analyses, LDL was found to be a protective factor for OA, while HDL still needs further study. Our results provide new biomarkers for preventive and therapeutic strategies for OA.

## Introduction

Degenerative joint diseases such as osteoarthritis (OA), the clinical manifestations are characterized by joint pain, restricted joint movement and bone rubbing sounds, affect patients’ quality of life seriously^1^. Globally, the age-standardized point prevalence and the annual incidence rate of OA in 2017 were 3754.2^2^ and 181.2 per 100,000 and has a wide range of factors such as age, obesity, inflammation, etc^3^. In recent years, studies have identified lipid biomarkers that may be associated with the development of OA, including triglycerides (TG), total cholesterol (TC), high-density lipoprotein (HDL), and low-density lipoprotein (LDL)^4^. For example, Puenpatom et al. found differences between TG and HDL in OA and non-OA populations^5^. Moreover, Garcia-Gil et al. found that levels of TG were associated with hand OA, whereas TC and LDL were not associated with hand OA^6^. Though these studies have explored the association between lipid biomarkers and OA, the association between the two is still unclear.

Existing methods for studying causal analysis contain propensity score matching (PSM), inverse probability weighting (IPW) and Mendelian randomization. PSM is mainly used to deal with selective bias in observational data, and IPW is more suitable for dealing with missing data or non-response. Among these methods, MR provides a way to estimate causal effects based on genetic information, which can better control for potential confounders and thus reduce bias and uncertainty. MR is a statistical method for analysing the causal relationship between exposure factors and outcomes by using instrumental variables (IV)^7^. George published the first MR article in 2003, suggesting that MR can provide insight into environmental determinants of disease and formalizing a research framework and study design for MR^8^. MR, which is based on whole-genome sequencing data, is effective in reducing bias, similar to RCT studies, and has been widely used in studies of causal relationships between exposure factors and outcomes^9^. Mendelian randomisation is one of the more widely used causal inference methods in epidemiology in recent years^10^.

The National Health and Nutrition Examination Survey (NHANES) is a population-based cross-sectional survey that is designed to collect information on the health and nutrition of the US population, containing demographic data and lifestyle and health and nutrition status information on participants, and has been used extensively in the study of morbidity factors^11,12^. In this study we screened participants with lipid markers from NHANES database and firstly combined cross-sectional study and MR study to explore the association between them from two dimensions, which makes the conclusions more reliable.

## Methods and materials

This study firstly examined the association between lipid biomarkers and OA using data from the NHANES. Then, in the framework of MR analysis, we performed two-sample MR based on published data from genome-wide association studies to further assess the causal association between lipid biomarker and OA.

### Study population in NHANES

In the cross-sectional study, data from a total of nine cycles of the NHANES database from 2003 to 2020 were used. Data on the presence or absence of osteoarthritis were obtained from survey documentation data. The data were first used to identify participants without osteoarthritis according to "Doctor ever said you had arthritis" and then to identify participants with osteoarthritis according to "Which type of arthritis". Please see the NHANES database for details of the populations that can be measured. (https://wwwn.cdc.gov/Nchs/Nhanes/2003-2004/MCQ_C.htm#Component_Description). Informed consent was obtained from all subjects in the National Health and Nutrition Examination Survey. Information for the assessment of OA was obtained through questionnaires and self-reports^13,14^. In a study conducted by March et al. the agreement between self-reported OA and clinically well-defined OA was 85%, indicating that OA can be accurately diagnosed in the majority of case reports^15^.

### Lipid biomarkers in NHANES

Lipid biomarkers include TG, TC, HDL and LDL. Lipid Biomarker tests are performed on a fasted state and serum samples are processed, stored and transported to the partner laboratory service for analysis. TG, TC, HDL, LDL units are all in mg/dl^16^. The measurement of TC and TG was performed with enzymatic assays, and the measurement of HDL was performed with immunoassays. LDL was calculated according to the Friedewald equation^17^. Detailed specimen collection and processing instructions are discussed in the NHANES Laboratory/Medical Technologists Procedures Manual. The NHANES quality control and quality assurance protocols meet the 1988 Clinical Laboratory Improvement Act mandates.

### Covariates used in NHANES

Refer to previous studies^18–20^, variables that may confound the association between lipid biomarkers and OA were collected. Information regarding demographics and lifestyle factors was collected by questionnaire, including age (years), sex (female, male), race/ethnicity (White, Mexican, Black, other), education (under high school, high school or equivalent, above high school), marital status (married, living with partner, separated, divorced, widowed, never married), smoking (never, former, now), alcohol intake (never, former, heavy, mild, moderate),cancer (no, yes),use of atherosclerosis conditions (no, unknown, yes). Health examination was performed in the mobile centers. Body mass index (BMI, kg/m^2^). Diabetes was defined as achieving a fasting glucose level of 126 mg/dL or reporting a previous diagnosis. Hypertension was defined as resting blood pressure (BP) persistently at or above 140/90 mmHg or reporting a previous diagnosis. Poverty is an index based on family income and federally defined poverty thresholds, reflecting income related to family needs^21^. Poverty ranges from 0 (no income) to 5 (greater than or equal to five times the federal poverty level)^22^. Physical activity is measured in MET (Metabolic equivalent, MET). MET (metabolic equivalent, MET) is the oxygen consumption required to maintain resting metabolism^23^. Based on energy expenditure in a quiet sitting position, it is a commonly used indicator to express the relative level of energy metabolism in various activities. The recommended MET values for work-related vigorous exercise, moderate exercise, walking or cycling, strenuous exercise, and moderate exercise were 8.0, 4.0, 4.0, 8.0, and 4.0, respectively. For each activity, physical activity was calculated in MET-min per week by multiplying the number of days by the average duration times the recommended MET and summing the resulting values to obtain an estimate of total physical activity^24^.

### Sources of MR

The MR study used two-sample MR. Exposure factors were TG, TC, HDL, LDL. GWAS data for TG, HDL and LDL are from the paper published by Richardson et al. in 2020^25^. TC data from the paper Borges CM et al. 2020^25^. Outcome factors for OA were derived from hospital-confirmed OA, containing 50,508 Sample sizes and 15,845,511 Single Nucleotide Polymorphisms (SNPs) sequenced by Zengini E et al. in 2018^26^. Informed consent was obtained from all subjects in the original genome-wide association studies. Detailed information is shown in Table 1.

**Table 1:** Detailed information on expose/outcome factor.

Expose/outcome	Year	Population	Sex	Sample size	Number of SNPs	Author	GWAS ID	PMID
TG	2020	European	Males and females	441,016	12,321,875	Richardson Tom	ieu-b-111	32203549
TC	2020	European	Males and females	115,078	12,321,875	Borges CM	met-d-Total_C	NA
HDL	2020	European	Males and females	403,943	12,321,875	Richardson Tom	ieu-b-109	32203549
LDL	2020	European	Males and females	440,546	12,321,875	Richardson Tom	ieu-b-110	32203549
OA	2018	European	Males and females	50,508	15,845,511	Zengini E	ebi-a-GCST005814	29559693

### Statistical analysis

The NHANES data analysis refers to the NHANES statistical tutorial and follows its complex multi-stage probability sampling with weighting of the sample. The weighting variable was chosen as wtsaf2yr, calculated as 1/9 * wtsaf2yr, and all analyses were performed under complex weighting. Continuous variables in normal distribution should be described as mean ± standard deviation (SD) or else reported as median (Range). Variance homogeneous and normal distributed continuous variables could be compared by student t-test, otherwise, the Mann–Whitney *U*-test or Kruskal–Wallis H-test should be used. Count data were statistically described by rates, and Poisson regression or Negative binomial regression was used for comparison between groups. Weight logistic regression models were used to test the associations of TG, TC, HDL, LDL with OA. All covariates were using the lowest quartile as the reference. Model 1 is weight logistic regression, the independent variable is each lipid biomarker and the dependent variable is OA; Model 2 was adjusted for age, sex, and race/ethnicity; Model 3 was further adjusted for age, sex, race/ethnicity, BMI, marital status, education, poverty, smoking, alcohol intake, hypertension, diabetes, cancer, physical activity and atherosclerosis. To better explore the association between lipid biomarkers and OA, logistic regression was conducted to explore the association with OA when lipids are used as quartiles.

MR was used to explore the causal relationship between lipid biomarkers and OA. All IV were selected using the same criteria. Exposure factors with genome-wide significance parameters were set to *P* < 5 × 10^–8^, the linkage disequilibrium parameter (r^2^) parameter was set to 0.001, and the genetic distance was set to 10 MB to screen for IV with no linkage effects. Association between lipid biomarker and OA was assessed using an inverse variance weighting (IVW) method as the main statistical method, theory has been described in previous studies^27,28^. Heterogeneity was examined using the IVW method and the MR-Egger method. Sensitivity analysis was performed using the leave-one-out method. Pleiotropy analysis was performed using the Egger-intercept method. Finally, the strength of association of the genetic instruments for each putative risk factor was quantified by the *F* statistic (*F* = β^2^/se^2^) for all SNPs, to assess the power of the SNPs^29^. All statistical analyses were performed using R software (Version 4.1.2; http://www.R-project.org, R Foundation for Statistical Computing, TUNA Team, Tsinghua University), the "nhanesR" package for NHANES data analysis and the "TwoSampleMR" package for MR analysis.

### Ethical approval

Informed consent was obtained from all subjects in the original genome-wide association studies and National Health and Nutrition Examination Survey, which were approved by NCHS Ethics Review Board. Therefore, per the guidelines of the XYZ Institutional Review Board, IRB assessment was not necessary.

## Results

### Lipid biomarkers and OA in NHANES

**Figure 1:**
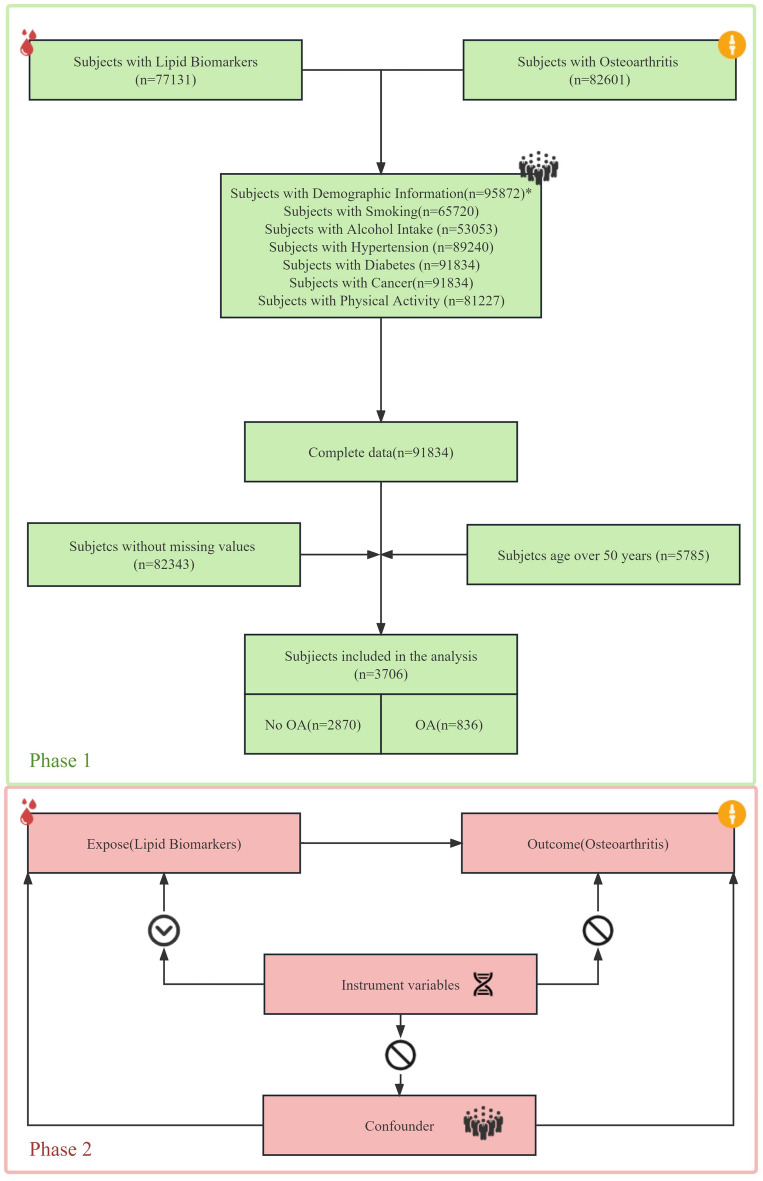
Study design overview.

Between 2003 and 2020, 82,601 participants were evaluated for OA and 77,131 participants were available for lipid biomarkers results, resulting in a total of 91,834 participants after combining all covariates. After removing participants with missing values, 9492 participants remained. 3706 participants were older than 50 years, of whom 836 had OA and 2870 did not have OA. After weighting, this represents 40,802,041 participants. The detailed process is shown in Fig. 1. The two groups were grouped according to whether they had OA or not, and there was a statistical difference (*P* < 0.05) between the two groups in age, BMI, race/ethnicity, marital status, alcohol intake, hypertension, cancer, atherosclerosis, HDL, LDL in Table 2. The results of univariate logistic showed that the OR (95% CI) for TG, TC, HDL and LDL were 1.00 (1.00, 1.00), 1.00 (1.00, 1.00), 1.01 (1.00, 1.01) and 0.99 (0.99, 1.00), respectively, with P values of 0.12, 0.08, 0.02 and < 0.001. The results of multifactorial logistic (model 3) showed that the OR (95% CI) for TG, TC, HDL, and LDL were 1.00 (1.00, 1.00), 1.00 (1.00, 1.00), 1.01 (1.00, 1.01), and 1.00 (0.99, 1.00), respectively, and the p-value was 0.62, 0.37, 0.049, and 0.049, respectively. The detailed results are shown in Table 3. When lipid biomarkers were divided into quartiles, the detailed results of the logistics results are shown in Table 3.

**Table 2:** Weighted selected characteristics of study population in female and male, NHANES (Weighted N = 40,804,313).

Characteristics	Total	No-OA	OA	*P* value
Sex, n (%)				
Female	1993 (49.76)	1676 (53.74)	317 (38.42)	Ref
Male	1713 (50.24)	1194 (46.26)	519 (61.58)	< 0.0001
Age (year), mean (SD)	61.80 (0.19)	60.81 (0.21)	64.6 1(0.36)	< 0.0001
BMI, mean (SD)	28.42 (0.13)	27.94 (0.14)	29.78 (0.26)	< 0.0001
Race/ethnicity, n (%)				
White	1946 (79.11)	1382 (76.61)	564 (86.24)	Ref
Mexican	462 ( 3.88)	396 (4.47)	66 (2.19)	< 0.0001
Black	624 ( 7.65)	521 (8.61)	103 (4.90)	< 0.0001
Other	674 ( 9.37)	571 (10.31)	103 (6.68)	< 0.0001
Marital status, n (%)				
Married	2282 (66.44)	1778 (66.57)	504 (66.05)	Ref
Living with partner	133 ( 3.22)	108 (3.48)	25 (2.49)	0.431
Separated	108 ( 1.61)	96 (1.94)	12 (0.67)	0.019
Divorced	510 (13.48)	397 (13.64)	113 (13.01)	0.975
Widowed	425 ( 9.16)	290 ( 7.96)	135 (12.58)	< 0.0001
Never married	248 ( 6.09)	201 (6.41)	47 (5.19)	0.316
Education, n (%)				
Under high school	768 (11.11)	637 (11.51)	131( 9.96)	Ref
High school or equivalent	837 (23.19)	636 (23.68)	201 (21.80)	0.002
Above high school	2101 (65.70)	1597 (64.81)	504 (68.24)	< 0.0001
Poverty, mean (SD)	3.51 (0.05)	3.52 (0.05)	3.45 (0.10)	0.43
Smoking, n (%)				
Never	1849 (50.86)	1455 (52.02)	394 (47.54)	Ref
Former	1312 (35.04)	982 (33.55)	330 (39.31)	0.026
Now	545 (14.10)	433 (14.44)	112 (13.15)	0.735
Alcohol intake, n (%)				
Never	488 (10.48)	379 (10.34)	109 (10.88)	Ref
Former	732 (15.24)	572 (15.48)	160 (14.57)	0.862
Mild	1625 (48.59)	1223 (47.07)	402 (52.92)	0.344
Moderate	463 (14.56)	358 (14.79)	105 (13.88)	0.912
Heavy	398 (11.13)	338 (12.32)	60 ( 7.74)	0.015
Hypertension, n (%)				
No	1549 (46.28)	1274 (49.29)	275 (37.68)	Ref
Yes	2157 (53.72)	1596 (50.71)	561 (62.32)	< 0.0001
Diabetes, n (%)				
No	3091 (87.65)	2405 (88.31)	686 (85.75)	Ref
Yes	615 (12.35)	465 (11.69)	150 (14.25)	0.295
Cancer, n (%)				
No	3154 (83.75)	2485 (85.47)	669 (78.85)	Ref
Yes	552 (16.25)	385 (14.53)	167 (21.15)	< 0.0001
Physical activity (MET/week), mean (SD)	3103.84 (101.91)	3149.90 (116.79)	2972.47 (192.50)	0.42
Atherosclerosis, n (%)				
No	974 (24.99)	883 (30.07)	91 (10.51)	Ref
Unknown	2728 (74.93)	1984 (69.85)	744 (89.43)	< 0.0001
Yes	4 (0.08)	3 (0.08)	1 (0.06)	0.328
TG (mg/dl), mean (SD)	123.50 (1.65)	122.10 (1.90)	127.51 (3.02)	0.12
TC (mg/dl), mean (SD)	201.47 (0.91)	202.39 (1.04)	198.84 (1.72)	0.07
HDL (mg/dl), mean (SD)	57.43 (0.48)	56.90 (0.55)	58.95 (0.75)	0.02
LDL (mg/dl), mean (SD)	119.33 (0.79)	121.07 (0.88)	114.39 (1.53)	< 0.001

**Table 3:** Weighted ORs (95% CIs) of the associations between lipid biomarkers and OA.

Characteristics	Model	Continuous		Q1	Q2		Q3		Q4	
		OR (95% CI)	*P* value		OR (95% CI)	*P* value	OR (95% CI)	*P* value	OR (95% CI)	*P* value
TG	Model 1	1.00 (1.00,1.00)	0.115	Ref	0.998 (0.729,1.367)	0.992	1.270 (0.967,1.669)	0.085	1.226 (0.918,1.637)	0.167
	Model 2	1.00 (1.00,1.00)	0.051	Ref	0.952 (0.690,1.314)	0.952	1.210 (0.898,1.630)	1.21	1.269 (0.927,1.738)	1.269
	Model 3	1.00 (1.00,1.00)	0.619	Ref	0.920 (0.654,1.293)	0.627	1.063 (0.776,1.457)	0.7	1.033 (0.741,1.440)	0.847
TC	Model 1	1.00 (1.00,1.00)	0.077	Ref	0.930 (0.723,1.197)	0.572	0.875 (0.695,1.102)	0.255	0.771 (0.586,1.015)	0.063
	Model 2	1.00 (0.99,1.00)	0.007	Ref	0.974 (0.747,1.269)	0.842	0.849 (0.663,1.087)	0.192	**0.679 (0.508,0.907)**	**0.009**
	Model 3	1.00 (1.00,1.00)	0.374	Ref	1.046 (0.804,1.361)	0.735	0.975 (0.746,1.273)	0.849	0.860 (0.628,1.177)	0.342
HDL	Model 1	1.01 (1.00,1.01)	0.015	Ref	1.135 (0.834,1.545)	0.418	1.128 (0.821,1.552)	0.454	1.385 (1.027,1.870)	0.033
	Model 2	1.00 (0.99,1.00)	0.538	Ref	0.952 (0.688,1.316)	0.763	0.842 (0.607,1.168)	0.3	0.908 (0.654,1.262)	0.564
	Model 3	1.01 (1.00,1.01)	**0.049**	Ref	1.062 (0.759,1.487)	0.721	1.062 (0.747,1.510)	0.735	1.409 (0.953,2.083)	0.085
LDL	Model 1	0.99 (0.99,1.00)	** < 0.001**	Ref	0.904 (0.670,1.220)	0.506	**0.672 (0.511,0.884)**	**0.005**	**0.579 (0.429,0.780)**	** < 0.001**
	Model 2	0.99 (0.99,1.00)	** < 0.001**	Ref	0.971 (0.704,1.340)	0.857	**0.726 (0.542,0.973)**	**0.033**	**0.582 (0.423,0.800)**	**0.001**
	Model 3	1.00 (0.99,1.00)	**0.049**	Ref	1.043 (0.759,1.432)	0.795	0.751 (0.547,1.033)	0.077	**0.713 (0.513,0.992)**	**0.044**

Model 1: no adjusted; Model 2: adjusted for age, sex, and race/ethnicity; Model 3: adjusted for all the covariates.

TG: Q1 [18,77], Q2 (77,108], Q3 (108,153], Q4 (153,399]; TC: Q1 [79,171], Q2 (171,198], Q3 (198,226], Q4 (226,463]; HDL: Q1 [21, 44], Q2 (44,53], Q3 (53,66], Q4 (66,173]; LDL: Q1 [15,94], Q2 (94,116], Q3 (116,141], Q4(141,375].

*TG* triglycerides, *TC* total cholesterol, *HDL* high density lipoprotein, *LDL* low density lipoprotein.

Significant values are in bold.

### Causal association between lipid biomarkers and OA in MR

The same statistical process was used for the causal analysis of lipid biomarkers and OA. The IVW results for TG showed an OR (95% CI) of 1.059 (0.969 to 1.157), TC showed an OR (95% CI) of 0.950 (0.851 to 1.061), HDL showed an OR (95% CI) of 0.936 (0.858 to 1.021), LDL showed an OR (95% CI) of 0.892 (0.797–0.998). The F-values are all greater than 10. Heterogeneity tests, sensitivity analysis, and pleiotropy analysis were all negative. According to the three assumptions of MR, there is a causal relationship between LDL and OA, and there is no causal relationship between TG, TC, HDL and OA. Detailed results are shown in Table 4.

**Table 4:** Mendelian randomization analysis of the main results.

Exposure	SNPs	Method	OR(95%CI)	*P*_Effect_	*P*_Heterogeneity_	*P*_Intercept_
TG	215	IVW	1.059 (0.969–1.157)	0.209	0.862	0.893
TC	48	IVW	0.950 (0.851–1.061)	0.366	0.761	0.929
HDL	247	IVW	0.936 (0.856–1.021)	0.133	0.971	0.243
LDL	126	IVW	0.892 (0.797–0.998)	0.046	0.676	0.864

*TG* triglycerides, *TC* total cholesterol, *HDL* high density lipoprotein, *LDL* low density lipoprotein, *IVW* inverse variance weighting.

## Discussion

OA is a common clinical degenerative condition. With an increasing proportion of obese and old people, OA brings a huge economic burden to society^30^. Early detection of risk factors for osteoarthritis can help in the prevention and treatment of the disease. In our study, we combined a cross-sectional study and MR to explore the relationship between lipid biomarkers and OA. The logistic regression results showed no association between TG, TC, and OA, but an association between HDL, LDL and OA. MR used a two-sample MR method, and the results showed no causal association between TG, TC, HDL and OA, but a causal association between LDL and OA (IVW results showed an OR value of 0.892 (0.797–0.998), *P*-value = 0.046).

By summarizing previous research, we found that our results are more reliable. We combined a cross-sectional study and MR to explore the relationship between lipid biomarkers and OA. By combining these two methods, we can effectively combine the advantages and disadvantages of the two methods. Based on the above results, we have found that HDL is a protective factor for OA, which should be paid enough attention and has some guiding sense for the clinic. At the same time, the control of lipid biomarkers should be strengthened to help the prevention and treatment of OA.

TG is the most abundant and most productive energy substance in the body and has been found to be closely associated with diseases such as coronary heart disease and diabetes^31^. Previous studies on the relationship between TG and OA are unclear; Zhou et al. found that the prevalence and incidence of knee OA increased by 9% and 5% respectively for a one-unit increase in TG, respectively^32^; Puenpatom et al. found high TG in people with OA in comparison to those without OA (47% vs. 32%)^5^; Askaria et al. found an association between TG and OA^33^. In contrast, our study did not find an association between TG and OA in the cross-sectional study, the same conclusion as that found by Zhang et al.^34^, who found no difference in TG between the OA and healthy groups, and Hindy et al. found no association between TG and OA^35^. To confirm the results of the cross-sectional study, further analysis that utilised MR showed that there was no causal relationship between TG and OA. Previously, Funck-Brentano et al. also used MR but found no causal relationship between TG and OA^36^, and Zengini et al. also found no causal relationship between TG and OA^26^. Combining the cross-sectional results with those of MR reveals that there is no relationship between TG and OA.

TC is a lipid-like substance found in blood lipoproteins and essential for cells. Some previous studies have examined the association between TG and OA^37^. Singh et al. found that the OA group had a higher proportion of high TC (32% vs. 24%) than did the control group^38^; Abdurhman et al. found that high levels of TC were associated with OA^22^; Zhang et al. found that levels of TC were higher in the OA group in comparison to the healthy group^34^. Meanwhile, Schwage et al. found no association between TC and OA, while Chingford found no association between TC and the incidence of hand OA^39^. In contrast, our study first used a cross-sectional study to find no association between TC and OA, and then combined it with MR to find no causal relationship between the two, thus supporting the conclusion that there is no association between TC and OA.

HDL is an anti-atherosclerotic lipoprotein that is synthesised mainly in the liver and transports cholesterol from extra-hepatic tissues to the liver for metabolism^39^. Our study showed HDL as a risk factor for OA in a cross-sectional study, which is the same as the findings of some of the previous studies. Pan et al. found an association between reduced HDL and a loss of medial tibial cartilage volume^40^, while Askaria et al. found an association between HDL and OA^33^; Zhang et al. found reduced levels of HDL in the OA group in comparison to the healthy group^34^; Puenpatom et al. found lower HDL in people with OA than in those without OA (44% vs. 38%)^5^. However, MR analysis shows no causal link between HDL and OA, which is the same finding as that of Hindy et al. and Schwage et al.^35,39^, who found no observed association between HDL and OA in their observational studies, while Funck-Brentano et al. used MR to find no causal relationship between HDL and OA^36^. The results of the cross-sectional study contradict the results of Mendelian randomisation, and further studies are needed to clarify the relationship between HDL and OA.

LDL is a cholesterol-rich lipoprotein. Kruisbergen et al. found that LDL activation of circulating monocytes was a risk factor for OA^41^; Oliviero et al. found higher levels of serum LDL in patients with OA in comparison to controls^42^; Mishra et al. found higher LDL in the OA group than in the control group^43^. However, the results of this type of study are contrary to the results of the present study. In the cross-sectional study, the logistic regression results showed that the OR for LDL was less than 1 and that the *P*-value was less than 0.05, whereby suggesting a association between LDL and OA, while the MR results showed an OR value (95% CI) of 0.892 (0.797–0.998). A heterogeneity test, sensitivity analysis, and pleiotropy analysis all showed negative results, which suggested a causal relationship between LDL and OA. The relationship between LDL and OA was demonstrated at two levels. Previously, George Hindy, E. Gill, Wang et al. using Mendelian randomisation, all found LDL to be a protective factor in OA^44,45^, consistent with the results of the present study, and suggested a corresponding possible mechanism by which LDL reduces APOA1 levels and serum amyloid A-induced arthritic inflammation in human primary chondrocytes and fibroblast-like synoviocytes.

However, there are still some shortcomings in this study. Due to the limitation of the data source, it is not possible to further analyse the type of OA, such as osteoarthritis of the knee, osteoarthritis of the hip, etc.; The OA data in the NHANES database is derived from questionnaires of patients' recollections, and there may be a certain recollection bias; Although the MR method was adopted in this study to investigate the causality of the two, but MR’s prerequisite is the existence of a linear relationship between the two, if not then MR is not applicable. Although we have combined cross-sectional studies and MR, prospective cohort data are still needed for verifying this, and basic experiments can be performed to explore the role of lipid markers in the development of OA.

## Conclusion

In summary, our study used cross-sectional studies and MR to demonstrate the relationship between lipid biomarkers and OA. LDL is a protective factor for OA. No relationship exists between TG, TC and OA, while HDL still needs to be proved by further studies. Our findings provide new biomarkers for preventive and therapeutic strategies for OA, but further studies on the underlying mechanisms are still needed.

## Data Availability

The datasets used and/or analysed during the current study available from the corresponding author on reasonable request.
